# Supplementary material for: Impaired Cu–Zn Superoxide Dismutase (SOD1) and Calcineurin (Cn) Interaction in ALS: A Presumed Consequence for TDP-43 and Zinc Aggregation in Tg SOD1G93A Rodent Spinal Cord Tissue
Source: Neurochem Res. 2018 Jan 3;44(1):228–33. doi: 10.1007/s11064-017-2461-z (PMC6345727; doi:10.1007/s11064-017-2461-z)
Supplement: Supplementary file 1 — Supplementary material 1 (PDF 1202 KB) [file 11064_2017_2461_MOESM1_ESM.pdf]

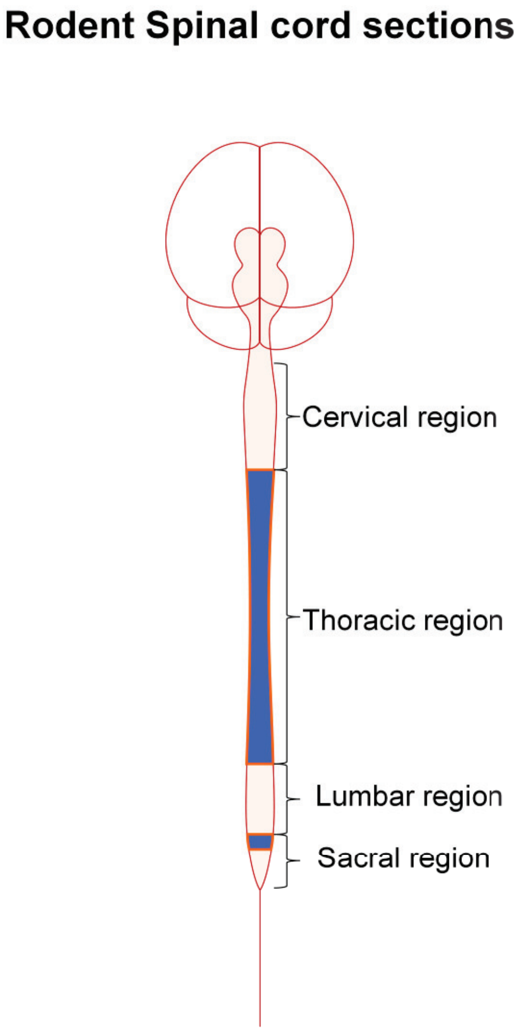

Fig-1S: The dissection template for rat spinal cord section.

TITLE : Impaired Cu-Zn superoxide dismutase (SOD1) and calcineurin (Cn) interaction in ALS: A presumed consequence for TDP-43 and zinc aggregation in Tg SOD1^{G93A} rodent spinal cord tissue.
Neurochemical Research

Corresponding author: Abdulbaki Agbas, aagbas@kcumb.edu

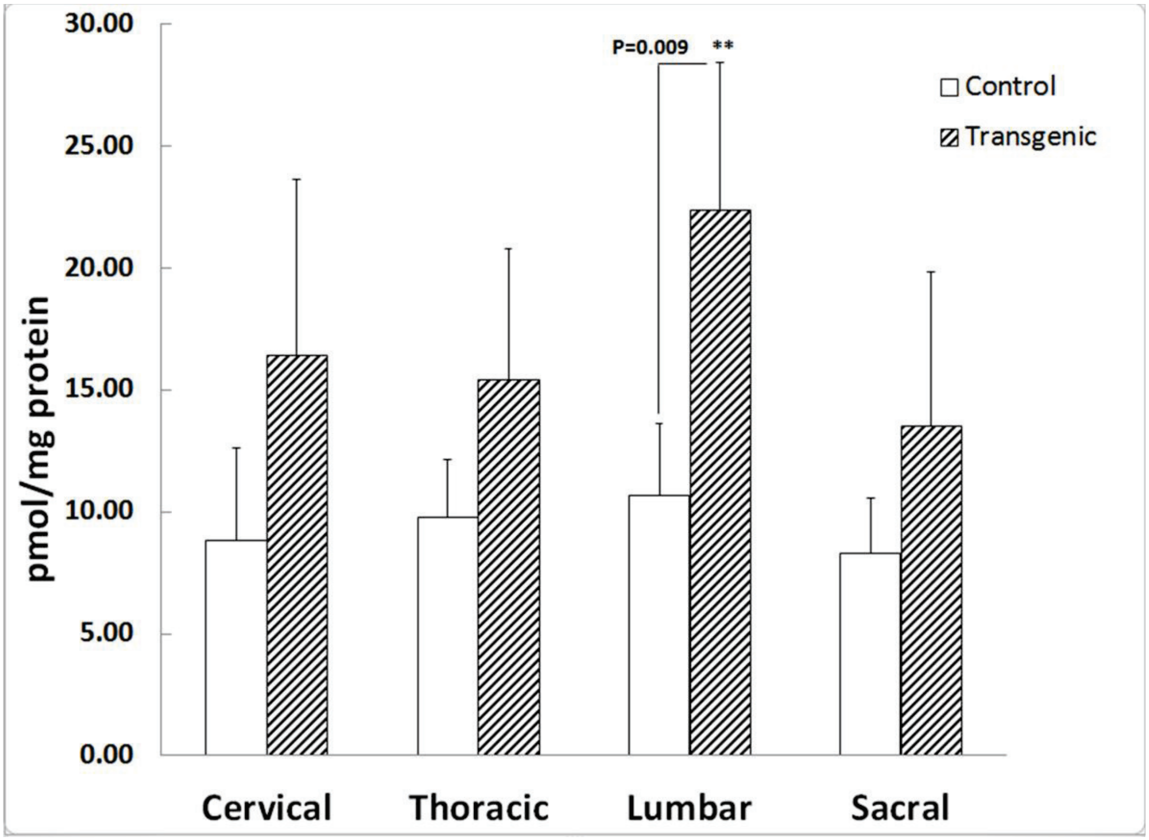

Fig. S4. SOD1^{G93A} Tg mouse spinal cord zinc concentrations. Total zinc concentrations were elevated in all sections of spinal cord. A statistical significance between two groups was obtained in lumbar region (P≤0.01) (n=6 per group). Data are ±SEM

TITLE : Impaired Cu-Zn superoxide dismutase (SOD1) and calcineurin (Cn) interaction in ALS: A presumed consequence for TDP-43 and zinc aggregation in Tg SOD1^{G93A} rodent spinal cord tissue.
Neurochemical Research

Corresponding author: Abdulbaki Agbas, aagbas@kcumb.edu

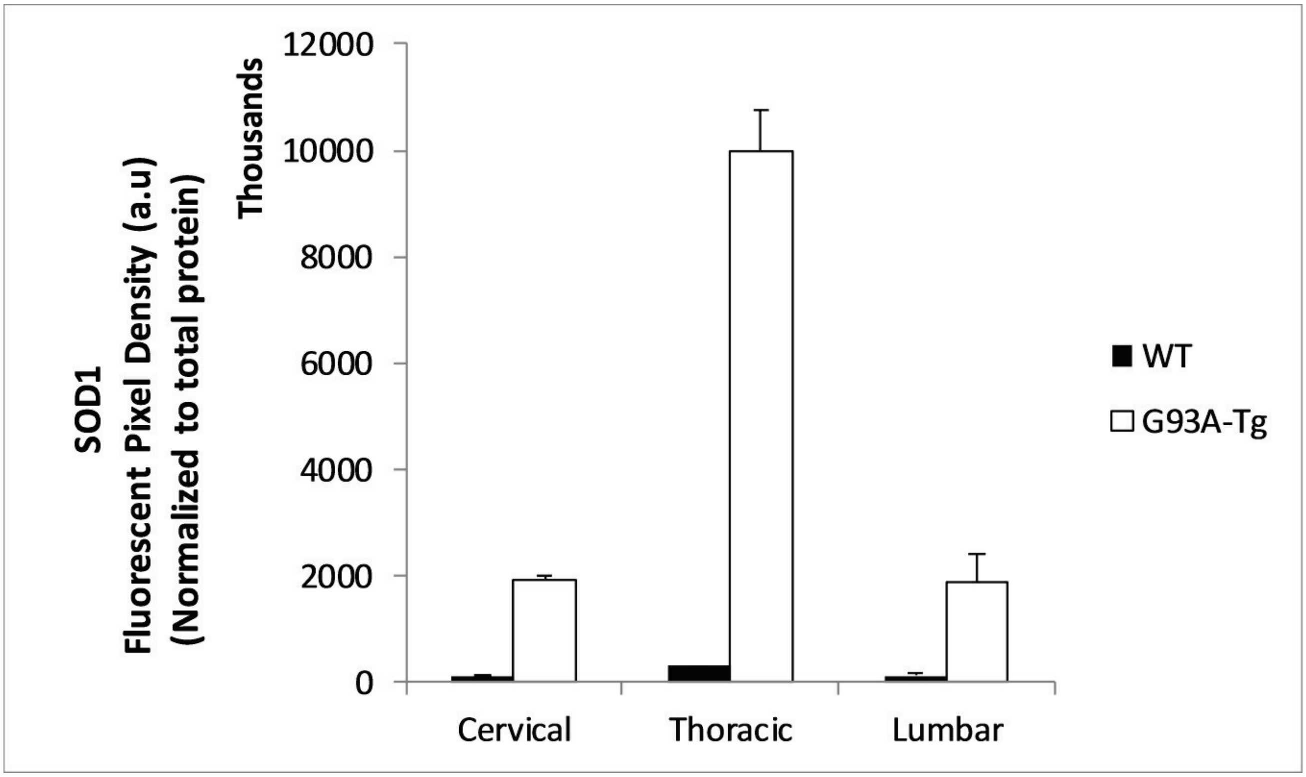

Fig. S3. SOD1 protein levels in all sections of spinal cord of SOD1G93A Tg rat. In all sections there were significant amount of SOD1 as expected. More SOD1 protein was observed in thoracic region as compared to others.

TITLE : Impaired Cu-Zn superoxide dismutase (SOD1) and calcineurin (Cn) interaction in ALS: A presumed consequence for TDP-43 and zinc aggregation in Tg SOD1^{G93A} rodent spinal cord tissue.
Neurochemical Research

Corresponding author: Abdulbaki Agbas, aagbas@kcumb.edu

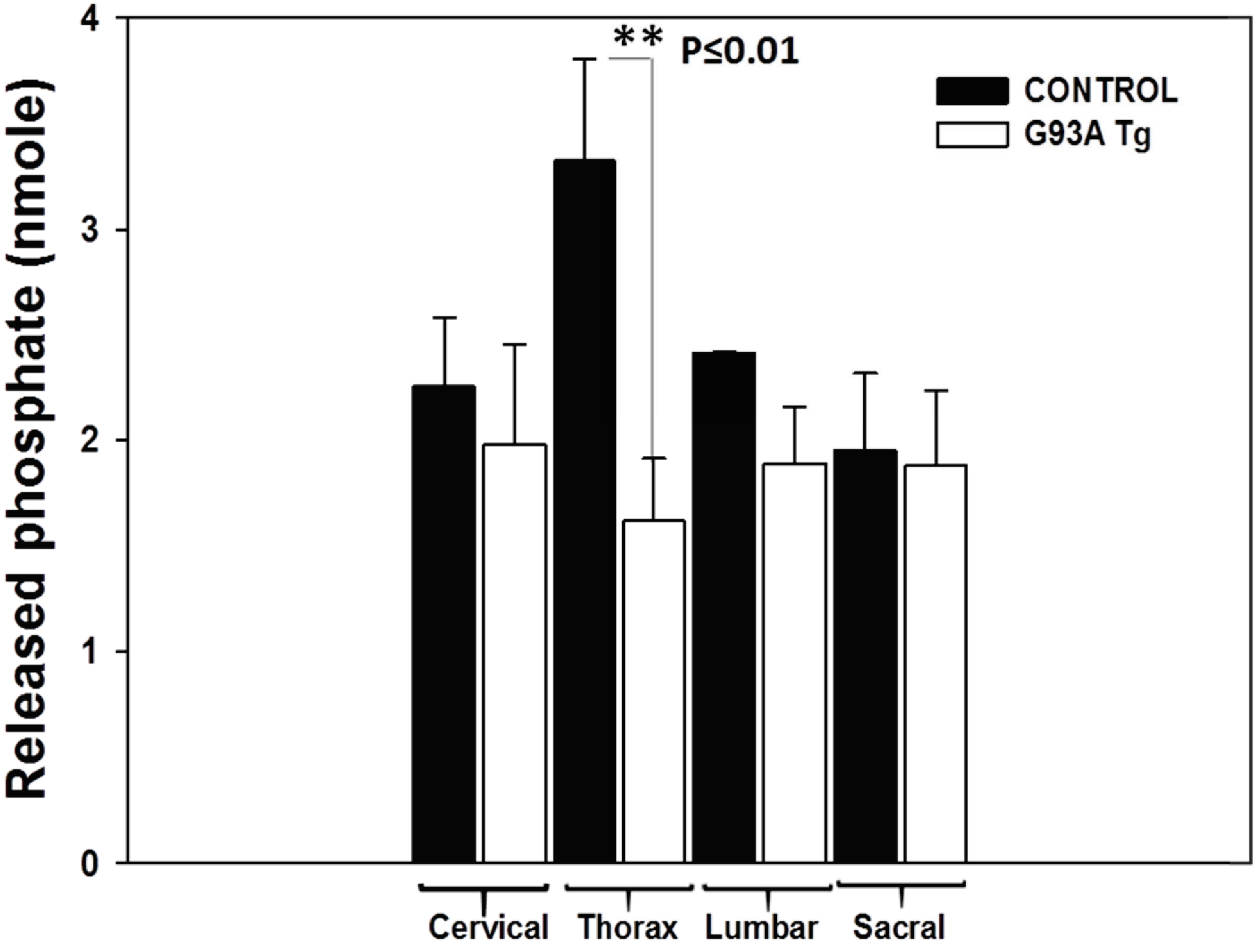

Fig. S2 Calcineurin enzyme activity levels in mice. Calcineurin enzyme activity levels were noticeably low in all spinal cord sections of the $SOD1^{G93A}$ Tg mice compared to that of the control. We have observed a significant difference in the thoracic region and it was statistically important ($P<0.01$) ($n=6$ per group). Data are mean±SEM

TITLE : Impaired Cu-Zn superoxide dismutase (SOD1) and calcineurin (Cn) interaction in ALS: A presumed consequence for TDP-43 and zinc aggregation in Tg $SOD1^{G93A}$ rodent spinal cord tissue.
Neurochemical Research

Corresponding author: Abdulbaki Agbas, aagbas@kcumb.edu

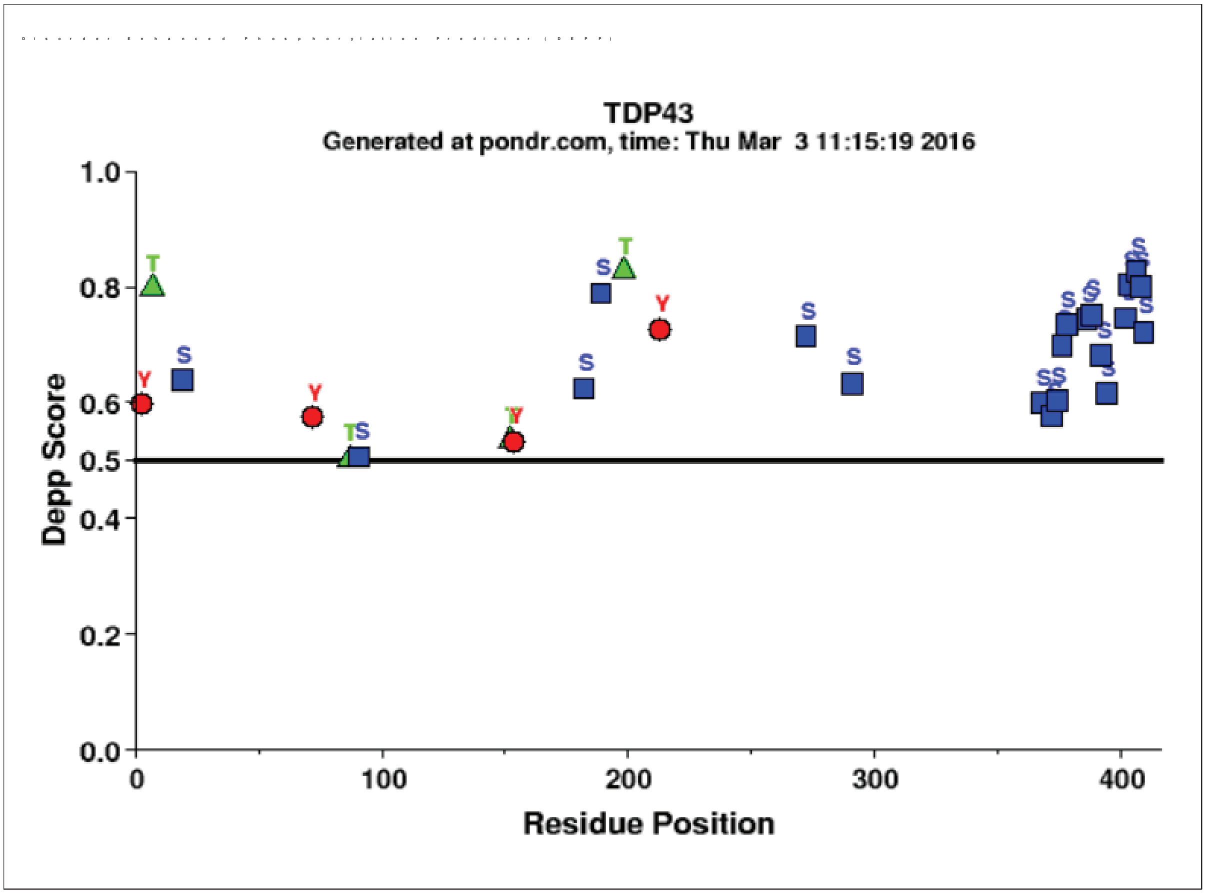

FIG.S5 PONDR analysis for TDP-43 predicted phosphorylation sites.

The VL3-BA predictor is a feedforward neural network that was trained on regions of 152 long regions of disorder that were characterized by various methods. The region close to C-terminus was identified as disordered sites for TDP-43 which is also most of the Serine amino acids are located.

www.molecularkinetics.com; main@molecularkinetics.com) under license from the WSU Research Foundation.
PONDR® is copyright ©1999 by the WSU Research Foundation, all rights reserved.

TITLE : Impaired Cu-Zn superoxide dismutase (SOD1) and calcineurin (Cn) interaction in ALS: A presumed consequence for TDP-43 and zinc aggregation in Tg SOD1^{G93A} rodent spinal cord tissue.
Neurochemical Research

Corresponding author: Abdulbaki Agbas, aagbas@kcumb.edu

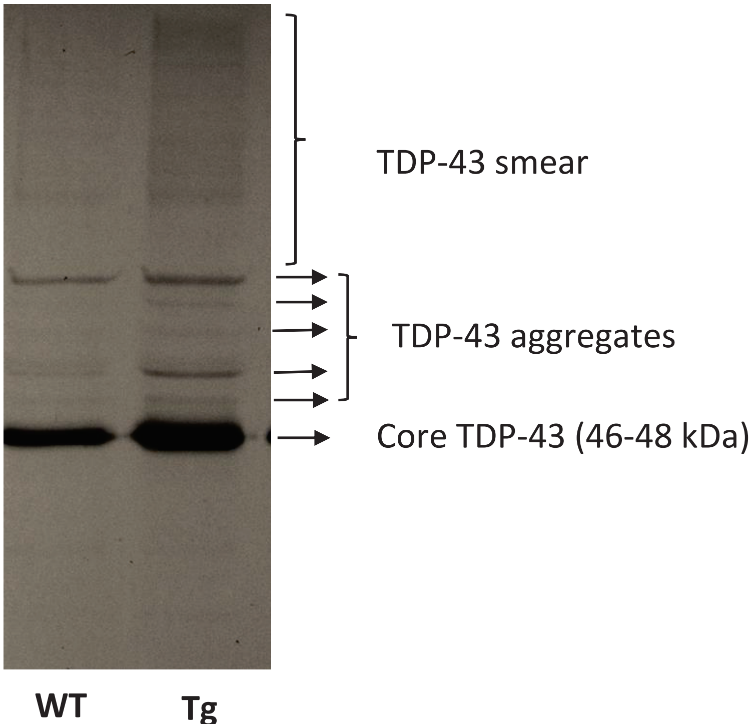

Fig. S6. Western blot analysis for TDP-43 aggregation in spinal cord thoracic region of rat. The smeary appearance of high molecular region may include TDP-43 aggregation. More distinct aggregated species appeared on above core protein.

TITLE : Impaired Cu-Zn superoxide dismutase (SOD1) and calcineurin (Cn) interaction in ALS: A presumed consequence for TDP-43 and zinc aggregation in Tg SOD1^{G93A} rodent spinal cord tissue.
Neurochemical Research

Corresponding author: Abdulbaki Agbas, aagbas@kcumb.edu
